# The Correlation Between Plasma CACNA2D1 Protein Concentration and the Severity of Coronary Heart Disease

**DOI:** 10.3390/jcdd13050214

**Published:** 2026-05-15

**Authors:** Le An, Yanhui Ren, Jin Yang, Zuowei Pei

**Affiliations:** 1 Department of Cardiology, Central Hospital of Dalian University of Technology, Dalian 116033, China; 18698706703@163.com (L.A.);; 2 Faculty of Medicine, Dalian University of Technology, Dalian 116024, China; 3 Department of Central Laboratory, Central Hospital of Dalian University of Technology, Dalian 116033, China; yjinjiny.cool@163.com; 4 Department of Geriatrics, Chengdu Fifth People’s Hospital Affiliated to Chengdu University of Traditional Chinese Medicine, Chengdu 611137, China

**Keywords:** coronary heart disease (CHD), calcium channel-encoding gene, CACNA2D1

## Abstract

Objective: The prevalence of coronary heart disease (CHD) continues to rise, and there is a lack of methods for early detection. To identify biomarkers for CHD, we analyzed the CACNA2D1 protein concentration in patients with different degrees of coronary artery stenosis to explore the correlation between plasma CACNA2D1 protein concentration and the severity of coronary artery stenosis. Methods: A total of 267 inpatients from the Department of Cardiology at Dalian Central Hospital who underwent coronary angiography were consecutively enrolled. According to the degree of stenosis, they were divided into four groups: minimal stenosis (70 cases), mild stenosis (68 cases), moderate stenosis (66 cases), and severe stenosis (63 cases). The baseline characteristics, clinical laboratory indicators, and CACNA2D1 protein concentration in blood samples of patients in each group were compared, and the correlations were analyzed. Results: As the degree of coronary artery stenosis worsened, plasma CACNA2D1 protein concentration in patients showed a gradual upward trend. The protein concentration was lowest in the mild stenosis group, at 37.68 ng/mL; it was 45.46 ng/mL in the mild-to-moderate stenosis group; it reached 55.22 ng/mL in the moderate stenosis group; and it was highest in the severe stenosis group, at 79.95 ng/mL. Conclusion: There is a correlation between plasma CACNA2D1 protein concentration and the degree of coronary artery stenosis, demonstrating that it has the potential to serve as a biomarker.

## 1. Introduction

The global prevalence of coronary heart disease (CHD) is projected to reach approximately 170 million by 2045 [1]. Although PCSK9 inhibitors and siRNA lipid-lowering therapies have significantly reduced LDL-C levels [2,3], the long-term rate of adverse events has not decreased in tandem [4,5], suggesting the existence of residual risk independent of lipid levels. Calcium homeostasis imbalance is a core component of atherosclerosis. Elevated calcium concentrations in vascular smooth muscle cells (VSMCs) can induce phenotypic transformation, migration, and calcification, directly promoting plaque growth and luminal loss [6]. However, current research on calcium channel-related genes remains focused on main channel subunits such as CACNA1C and CAV1, while insufficient attention is paid to auxiliary subunits.

The α2δ-1 auxiliary subunit encoded by CACNA2D1 is an essential component of voltage-gated calcium channels, which play pivotal roles in cellular physiology, including the regulation of vascular smooth muscle contraction, intracellular Ca^2+^ concentration, and signal transduction [7,8]. Mutations or altered expression of genes encoding calcium channel subunits have been closely associated with the initiation and progression of coronary artery disease. Variants in CACNA2D1 have already been linked to Brugada syndrome, short-QT syndrome, and hypertension-related myocardial injury [9,10,11]. Functional studies further demonstrate that the R217A point mutation completely eliminates pregabalin binding to α2δ-1, leading to the loss of nociceptive modulation [12]. This indicates that gene dosage or sequence variation in CACNA2D1 has significant biological effects.

Nevertheless, whether this gene influences coronary atherosclerotic burden through altered gene dosage (copy number variation, CNV) or expression levels remains systematically unexplored. In this study, we enrolled patients across the entire spectrum of stenosis severity and combined coronary imaging examination with ELISA-based quantification of plasma CACNA2D1 protein. Our primary objectives are to determine whether CACNA2D1 protein levels are associated with the degree of coronary stenosis and whether the protein has the potential to serve as a predictive biomarker of residual cardiovascular risk in CHD. The findings may provide observational evidence for the association between calcium channel auxiliary subunits and atherosclerotic severity, and may inform future hypothesis-driven research.

## 2. Materials and Methods

### 2.1. Study Participants

This study consecutively enrolled unrelated Chinese adults who were hospitalized in the Department of Cardiology at the Affiliated Central Hospital of Dalian University of Technology and underwent coronary imaging examination from October 2024 to October 2025 as subjects.

Inclusion criteria: Patients hospitalized in the Department of Cardiology of our hospital who had previously been confirmed to meet the diagnostic criteria for coronary heart disease (CHD) by coronary angiographic imaging, or patients with suspected CHD due to chest pain who underwent coronary angiography or coronary CT examination and had complete clinical data.

Exclusion criteria: Patients with incomplete clinical or coronary angiographic imaging data; patients with acute myocardial infarction; patients with a history of coronary angiography showing chronic total occlusion (CTO) lesions or a history of coronary artery bypass grafting (CABG); patients with heart function class III or above or those who had undergone heart transplantation; patients with moderate-to-severe anemia; patients in the active phase of inflammatory diseases; patients with severe liver or kidney dysfunction; patients with malignant tumors or hematologic diseases; patients with a history of surgical operations within the past three months. The diagnostic criteria for CHD were as follows: stenosis of one or more main coronary arteries > 50%. Coronary stenosis severity was assessed by two independent interventional cardiologists (for CAG) or a senior cardiac imaging specialist (for coronary CT). For coronary CT, stenosis was visually estimated and cross-validated against CAG when available. When inter-observer consistency is high, the result is deemed valid. Inconsistent cases are adjudicated by a third senior expert. All stenosis measurements were expressed as percentage luminal diameter reduction using a unified visual estimation method to ensure comparability across imaging modalities.

**Figure 1:**
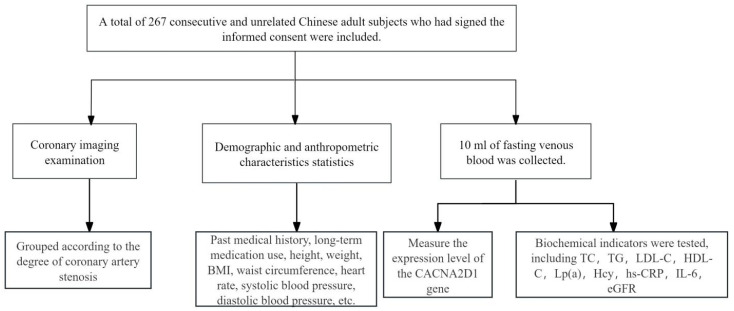
The flowchart of this study. BMI, body mass index; TC, total cholesterol; TG, triglyceride; LDL-C, low-density lipoprotein cholesterol; HDL-C, high-density lipoprotein cholesterol; Lp(a), lipoprotein (a); Hcy, homocysteine; hs-CRP, high-sensitivity C-reactive protein; IL-6, interleukin-6; eGFR, estimated glomerular filtration rate.

In this study, the degree of coronary artery stenosis was used as an indicator to assess the severity of CHD. The control group consisted of patients with stenosis < 50% and no clinical diagnosis of CHD, stratified according to minimal stenosis (≤25%) and mild stenosis (25–50%). The case group was stratified according to the degree of stenosis: 50–70% and >70% (Figure 1).

The distribution of the groups was as follows:

- Minimal stenosis group: 70 cases.
- Mild stenosis group: 68 cases.
- Moderate stenosis group: 66 cases.
- Severe stenosis group: 63 cases.

All participants provided informed consent.

### 2.2. Demographic and Anthropometric Characteristics

We recorded the gender, age, resting heart rate, and comorbidities such as hypertension and hyperglycemia for each subject, along with their smoking history and lipid-lowering medication use. Height and weight were measured twice. Subjects were barefoot and wearing light clothing, and the average of the two measurements was taken. Body Mass Index (BMI) = weight (kg) ÷ height^2^ (m^2^). The diagnosis of hypertension was based on the World Health Organization (WHO) criteria [13], and the diagnosis of diabetes was based on the American Diabetes Association (ADA) criteria [14]. On the first morning of hospitalization, fasting venous blood samples were collected from the patients and immediately sent to the clinical laboratory for the following serum biochemical tests: triglyceride (TG), total cholesterol (TC), low-density lipoprotein cholesterol (LDL-C), very-low-density lipoprotein cholesterol (VLDL-C), high-density lipoprotein cholesterol (HDL-C), lipoprotein (a) [Lp(a)], high-sensitivity C-reactive protein (hs-CRP), and homocysteine. All remaining blood samples were properly stored according to standard procedures for subsequent CACNA2D1 protein assays.

### 2.3. Measurement of CACNA2D1 Protein Concentration and IGFBP7 in Blood Samples

A commercial human voltage-dependent calcium channel α2δ-1 subunit (CACNA2D1) sandwich ELISA kit was used to measure the protein concentration of CACNA2D1 (Jiangsu Enzyme Immunoassay Industrial Co., Ltd., Changzhou, China). The sample concentration is expressed in ng/mL. For samples below the detection limit, a value of 0.05 ng/mL was assigned for statistical analysis. This detection method has the advantages of high specificity, high sensitivity, good reproducibility, simple operation, wide applicability, and reliable results, making it suitable for measuring the CACNA2D1 protein concentration in blood samples in this study. The CACNA2D1 content in all samples was measured twice, and the average of the two measurements was used for statistical analysis.

Plasma human insulin-like growth factor-binding protein-7 (IGFBP7) concentrations were measured using a commercially available human IGFBP7 ELISA research kit (Jiangsu Enzyme Immunoassay Industrial Co., Ltd., Changzhou, China), and the results were expressed in ng/mL. Samples below the limit of detection were assigned a value of 0.05 ng/mL for statistical analysis. All samples were assayed in duplicate.

### 2.4. Statistical Analysis of Data

Statistical analyses were performed using IBM SPSS Statistics 27 and GraphPad Prism 10 software. The Kolmogorov–Smirnov test was applied to assess whether the data were normally distributed. Data with a normal distribution were expressed as the mean ± standard deviation, and comparisons between two groups were made using the *t*-test. Data with a skewed distribution were expressed as median (interquartile range), and comparisons between two groups were made using the rank-sum test. Count data were expressed as numbers (n) and percentages (%), and comparisons were made using the chi-square test. The Spearman correlation coefficient and non-parametric tests (such as the Mann–Whitney U test or Kruskal–Wallis H test) were used to evaluate the correlation between the protein concentration of CACNA2D1 and clinical parameters such as LDL-C and homocysteine. Binary logistic regression analysis was used to evaluate the correlation between the degree of coronary artery stenosis and the protein concentration of CACNA2D1. The level of statistical significance was set at *p* < 0.05.

## 3. Results

### 3.1. Baseline Characteristics

A total of 267 suspected coronary heart disease (CHD) patients with assessable stenosis confirmed by coronary imaging examination were consecutively included. Table 1 summarizes the baseline data of all participants.

Overall comparisons showed that gender composition, smoking status, statin use rate, and total cholesterol (TC) levels were significantly unbalanced as the stenosis severity increased. In contrast, age, BMI, diabetes, hypertension, eGFR, and triglyceride levels were evenly distributed among the four groups.

### 3.2. Comparison and Analysis of Biochemical Indicators

A comparative analysis of biochemical indicators among different groups of coronary artery stenosis degrees was conducted (Table 2). The results showed significant differences among groups in low-density lipoprotein cholesterol (LDL-C) and homocysteine levels, while no statistical difference was found in lipoprotein (a) [Lp(a)] levels.

It is worth noting that the results of this study showed no significant correlation between Lp(a) levels and the degree of vascular stenosis, which is contradictory to the conclusions reported in several previous studies [15,16]. A variety of potential factors may have led to the emergence of this discrepancy.

### 3.3. Plasma CACNA2D1 Protein Concentration in Patients with Different Degrees of Coronary Artery Stenosis

**Figure 2:**
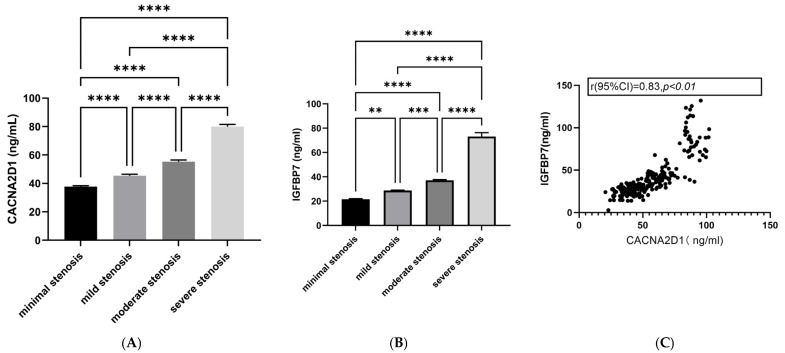
Bar chart of CACNA2D1 protein across different stenosis severity groups. (**A**) Comparison of CACNA2D1 protein concentration among different coronary artery stenosis severity groups (bar chart). (**B**) Comparison of insulin-like growth factor binding protein 7 (IGFBP7) protein concentration among different coronary artery stenosis severity groups (bar chart). (**C**) Correlation analysis between CACNA2D1 protein concentration and IGFBP7 protein concentration (scatter plot). ** *p* < 0.01, *** *p* < 0.001, and **** *p* < 0.0001.

Plasma CACNA2D1 protein concentration in patients with different degrees of coronary artery stenosis is shown in Figure 2. The results indicate that there is a significant positive correlation between the degree of coronary artery stenosis and the protein concentration of CACNA2D1 in patients with coronary atherosclerotic heart disease (CHD). The concentration trend of the CACNA2D1 protein is completely consistent with that of insulin-like growth factor binding protein 7 (IGFBP7). Furthermore, the scatter plot shows that the plasma CACNA2D1 protein concentration and IGFBP7 are significantly positively correlated.

### 3.4. Binary Logistic Analysis of the Degree of Stenosis and Plasma CACNA2D1 Protein Concentration

To explore the impact of plasma CACNA2D1 protein concentration on the degree of disease-related stenosis, this study used the degree of stenosis as the dependent variable and the plasma CACNA2D1 protein concentration as the independent variable. IBM SPSS Statistics 27 software was employed to conduct binary logistic regression analyses separately between the minimal and mild stenosis groups, the moderate and severe stenosis groups, and the case and control groups (Table 3).

CACNA2D1 protein concentration was a significant positive predictor of stenosis severity in unadjusted binary logistic regression. Specifically, a higher protein concentration is associated with an increased risk of more severe stenosis. This finding provides important evidence supporting the understanding of vascular stenosis at the molecular biological level.

### 3.5. Correlation Analysis: Protein CACNA2D1 Concentration and Associated Factors

Spearman correlation analysis was used to explore the associations between the mean CACNA2D1 protein concentration and various lipid metabolism indicators. The results (Table 4) showed that the mean CACNA2D1 protein concentration was significantly positively correlated with low-density lipoprotein cholesterol levels and also had a significant positive correlation with homocysteine levels. However, there was no significant correlation between the CACNA2D1 protein concentration and Lp(a) classification.

In addition, low-density lipoprotein cholesterol had no significant correlation with Lp(a) classification or homocysteine, and homocysteine also had no significant association with Lp(a) classification.

## 4. Discussion

In this study, coronary imaging examination was employed to show that plasma CACNA2D1 protein concentration increased gradually with the severity of stenosis. This finding implies that CACNA2D1 may be related to the severity of coronary stenosis; however, whether it contributes to disease progression remains to be clarified.

Among the 267 participants, sex, smoking status, and statin use rate were unbalanced across the four groups. The stepwise increase in statin use paralleled stenosis severity, reflecting more aggressive lipid-lowering therapy in patients perceived as high-risk—a classic example of confounding by indication. In contrast, age, BMI, diabetes, hypertension, eGFR, and triglycerides were evenly distributed and are therefore unlikely to act as important confounders. LDL-C is a major driver of atherogenesis; its deposition and subsequent oxidation in the arterial wall trigger inflammation, promote foam cell formation, and accelerate plaque initiation and progression [17,18]. Lp(a) levels correlate positively with coronary stenosis, and values ≥ 150 nmol/L are significantly associated with disease progression, making Lp(a) a robust predictor of coronary artery disease [19,20]. In the present cohort, both LDL-C and Lp(a) rose stepwise across groups, but only the LDL-C trend reached statistical significance. The absolute Lp(a) increment in the severe-stenosis group did not achieve *p* < 0.05, probably because of limited power; confirmation in an independent cohort of ≥400 subjects is warranted. Homocysteine differed significantly among groups, consistent with the established hyper-Hcy–endothelial injury–thrombosis pathway [21,22].

Bar plots of CACNA2D1 protein concentration showed a stepwise increase in plasma CACNA2D1 protein concentration across stenosis grades. Binary logistic regression further confirmed that CACNA2D1 protein concentration is associated with the severity of coronary narrowing. These findings provide observational evidence indicating that this protein may be a marker associated with disease severity.

Spearman analysis revealed that the CACNA2D1 protein concentration was positively correlated with both LDL-C and homocysteine. This suggests that CACNA2D1 may serve as a candidate biomarker for cardiovascular risk stratification. Causal relationships and therapeutic implications require validation in prospective and mechanistic studies. IGFBP7 is a well-established biomarker of coronary atherosclerosis [23] and adds prognostic value for incident and recurrent cardiovascular events [24]. A scatter plot of the plasma CACNA2D1 protein concentration versus the IGFBP7 concentration yielded a correlation coefficient of 0.83, implying that the two molecules may participate in a common pathobiological axis driving atherosclerotic progression. However, within the severe stenosis group, this association was attenuated. This attenuation may partly reflect the higher medication burden in severe cases, including calcium channel blockers, which modulate calcium channel activity and may confound the association.

The precursor protein encoded by the CACNA2D1 gene is cleaved to form the α2 and δ subunits, which together constitute the accessory subunit of the voltage-gated calcium channel complex. Calcium channels mediate the influx of calcium ions into cells during membrane polarization, which is crucial for maintaining normal cellular physiological functions. In vascular smooth muscle cells, the activation of calcium channels leads to cell contraction, causing vascular constriction. In endothelial cells, the regulation of calcium channels is closely related to the release of vasodilators. Additionally, calcium channels participate in intracellular signaling pathways, affecting processes such as cell proliferation, apoptosis, and inflammatory responses [25,26].

In atherosclerosis, CACNA2D1 may exert its effects through multiple mechanisms. It should be emphasized that the following mechanisms are speculative interpretations derived from cross-sectional observational data and require extensive validation through cellular experiments and animal models.

First, abnormal proliferation and migration of vascular smooth muscle cells are key drivers of coronary atherosclerotic stenosis [27]. Studies have shown that knockdown of CACNA2D1 in colon cancer inhibits cell proliferation and migration and alters the cellular microenvironment by regulating specific signaling pathways [28]. This suggests that CACNA2D1 may influence vascular smooth muscle cell behavior through similar mechanisms and participate in the progression of coronary artery stenosis. Second, vascular endothelial dysfunction is an early marker of coronary atherosclerosis [29]. Immunofluorescence double staining confirmed the colocalization of CACNA2D1 and CD31 in mouse cardiac endothelial cells. Silencing CACNA2D1 ameliorated Ang II-induced microvascular endothelial cell injury [11]. This indicates that abnormal CACNA2D1 protein levels may impair endothelial calcium channel function, leading to endothelial dysfunction and promoting atherosclerotic development. Third, inflammation is central to the initiation, progression, and plaque rupture of coronary atherosclerosis [30]. Research has shown that CACNA2D1 can regulate fibroblast activation and the release of inflammatory factors via the NF-κB signaling pathway in the tumor microenvironment [28]. Since NF-κB is a core transcription factor for inflammatory responses in atherosclerosis, the “CACNA2D1-Ca2+-NF-κB-inflammatory microenvironment” regulatory mechanism revealed in colon cancer is highly similar in atherosclerosis, despite the different pathological context. Therefore, it is speculated that abnormal CACNA2D1 protein levels may regulate macrophage function and exacerbate inflammatory responses by affecting calcium channel activation, thereby driving the progression of atherosclerotic lesions.

This study has several limitations. First, the control group consisted of symptomatic patients undergoing coronary evaluation rather than healthy controls. Therefore, the observed CACNA2D1 protein levels in this group may not reflect the true population normals, and selection bias cannot be excluded. Second, the small sample size of this study may limit the statistical power of the analysis. Additionally, all participants in this study were Chinese Han individuals who had lived in Dalian for a long time, and the results may not be applicable to other regions or ethnic groups. We acknowledge that the observed association between CACNA2D1 and stenosis severity was not adjusted for baseline imbalances (e.g., sex, smoking, statin use, and total cholesterol) due to the limited sample size. Therefore, this predictor should be interpreted as an unadjusted association rather than an independent causal factor. Although this study indicates that CACNA2D1 is associated with the severity of coronary artery stenosis in patients with coronary heart disease, the specific mechanisms of action have not yet been fully elucidated and require further investigation into its intracellular signaling pathways and interactions with other genes and proteins.

## 5. Conclusions

This study analyzed the CACNA2D1 protein concentration in patients with different degrees of coronary artery stenosis and found that the plasma CACNA2D1 protein concentration is positively correlated with the degree of stenosis. It indicates that plasma CACNA2D1 protein concentration is associated with the severity of coronary stenosis, and its role in disease progression deserves further investigation.

## Figures and Tables

**Table 1:** Comparison of Baseline Clinical Characteristics Among Groups.

		N	Minimal Stenosis N = 70	Mild Stenosis N = 68	Moderate Stenosis N = 66	Severe Stenosis N = 63	x^2^/H	*p*
Gender, n (%)	Men	101	35 (34.7)	33 (32.7)	21 (20.8)	12 (11.9)	18.183	*p* < 0.001
	Women	166	35 (21.1)	35 (21.1)	45 (27.1)	51 (30.7)		
Age (years)			63.5 (56.75, 70.00)	68 (61.25, 71.75)	65 (59.00, 70.00)	66 (57.00, 70.00)	4.515	0.21
BMI (kg/m^2^)			26.12 (23.84, 27.84)	25.63 (25.63, 29.39)	26.15 (24.51, 27.69)	25.39 (23.51, 29.30)	0.534	0.91
Smoke, n (%)	Yes	63	12 (19.0)	12 (19.0)	14 (22.2)	25 (39.7)	12.203	0.007
	No	204	58 (28.4)	56 (27.5)	52 (25.5)	38 (18.6)		
Diabetes, n (%)	Yes	84	18 (21.4)	17 (20.2)	22 (26.2)	27 (32.1)	6.290	0.098
	No	183	52 (28.4)	51 (27.9)	44 (24)	36 (19.7)		
Hypertension, n (%)	Yes	86	24 (27.9)	22 (25.6)	19 (22.1)	21 (24.4)	0.529	0.91
	No	181	46 (25.4)	46 (25.4)	47 (26)	42 (23.2)		
Statins, n (%)	Yes	238	54 (22.7)	59 (24.8)	63 (26.5)	62 (26.1)	19.116	*p* < 0.001
	No	29	16 (55.2)	9 (31.0)	3 (10.3)	1 (3.4)		
eGFR (mL/min/1.73 m^2^)			94.35 (83.02, 105.08)	84.37 (72.91, 100.99)	91.06 (75.24, 99.79)	88.75 (65.21, 100.63)	7.156	0.067
TC (mmol/L)			4.51 (3.50, 5.11)	3.80 (4.45, 5.16)	4.23 (3.54, 5.53)	5.23 (4.54, 7.47)	25.014	*p* < 0.001
TG (mmol/L)			1.51 (1.03, 2.78)	1.42 (0.93, 2.19)	1.56 (1.09, 2.18)	1.5 (1.10, 2.25)	1.599	0.66

BMI, body mass index; TC, total cholesterol; TG, triglyceride; eGFR, estimated glomerular filtration rate.

**Table 2:** Analysis of Differences in Biochemical Indicators Among Groups.

	Minimal Stenosis	Mild Stenosis	Moderate Stenosis	Severe Stenosis	H/x^2^	*p*
LDL-C (mmol/L)	2.16 (1.6, 2.77)	2.34 (1.77, 2.79)	2.10 (1.56, 3.18)	3.26 (2.77, 3.84)	58.870	*p* < 0.001
Hcy (umol/L)	10.37 (8.72, 12.90)	11.68 (9.42, 14.42)	11.45 (9.43, 14.05)	11.65 (9.73, 14.38)	10.354	0.016
Lp(a) (mg/mL)					4.957	0.85
<100	24 (24.0)	25 (25.0)	22 (22.0)	29 (29.0)		
100–300	33 (27.7)	30 (25.2)	33 (27.7)	23 (19.3)		
300–500	7 (28.0)	8 (32.0)	6 (24.0)	4 (16.0)		
>500	6 (26.1)	5 (21.7)	5 (21.7)	7 (30.4)		

There were differences in the distribution of LDL-C and Hcy among the groups, while there was no statistically significant difference in the distribution of LP(a) among the groups. *p* values were calculated using Kruskal–Wallis H test for continuous variables and χ^2^ test for categorical variables. LDL-C: low-density lipoprotein cholesterol; Hcy: homocysteine; Lp(a): lipoprotein (a).

**Table 3:** The results of binary logistic regression test (unadjusted).

Group	B	Wald χ^2^	df	Exp(B)	The 95% Confidence Interval for EXP(B)	
					Lower Limit	Upper Limit
Minimal and Mild	0.116	19.249	1	1.123	1.066	1.183
	−4.816	19.088	1	0.008		
Moderate and Severe	0.125	35.47	1	1.133	1.087	1.181
	−8.43	35.9	1			
control and cases	0.139	64.159	1	1.149	1.111	1.189
	−7.205	68.012	1	0.001		

All models are unadjusted.

**Table 4:** Multivariate correlation analysis.

	CACNA2D1 (ng/mL)	LDL-C (mmol/L)	Lp(a) (mg/mL)	Hcy (μmol/L)
*CACNA2D1* (ng/mL)	1			
LDL-C (mmol/L)	0.402 **	1		
Lp(a) (mg/mL)	−0.046	−0.091	1	
Hcy (μmol/L)	0.139 *	0.029	−0.023	1

LDL-C: low-density lipoprotein cholesterol; Hcy: homocysteine; Lp(a): lipoprotein (a). * *p* < 0.05, ** *p* < 0.01.

## Data Availability

The original contributions presented in the study are included in the article; further inquiries can be directed to the corresponding author.
